# Molecular and Clinical Characteristics of Primary Pulmonary Lymphoepithelioma-Like Carcinoma

**DOI:** 10.3389/fmolb.2021.736940

**Published:** 2021-10-25

**Authors:** Ying Fan, Qianyun Shan, Jiali Gong, Jing Qin, Hongyang Lu

**Affiliations:** ^1^ Zhejiang Key Laboratory of Diagnosis and Treatment Technology on Thoracic Oncology (Lung and Esophagus), Cancer Hospital of the University of Chinese Academy of Sciences (Zhejiang Cancer Hospital), Hangzhou, China; ^2^ Department of Thoracic Medical Oncology, Cancer Hospital of the University of Chinese Academy of Sciences (Zhejiang Cancer Hospital), Hangzhou, China; ^3^ Institute of Basic Medicine and Cancer (IBMC), Chinese Academy of Sciences, Hangzhou, China; ^4^ The First Clinical Medical College, Wenzhou Medical University, Wenzhou, China; ^5^ The Second Clinical Medical College, Zhejiang Chinese Medical University, Hangzhou, China

**Keywords:** PPLELC, clinical characteristics, molecular characteristics, immunotherapy, PD-L1, TMB

## Abstract

**Objectives:** Primary pulmonary lymphoepithelioma-like carcinoma (PPLELC) is an extremely rare subtype of non-small cell lung cancer (NSCLC). Currently, there are no established treatment protocols due to rarity of the cancer. Thus, this study aimed to explore the molecular and clinical characteristics of PPLELC.

**Study design and setting:** Data from patients with PPLELC who were admitted to Zhejiang Cancer Hospital from August 2009 to September 2020 were retrospectively collected. Next-generation sequencing was performed to obtain a genomic profile and tumor mutation burden (TMB) value of patients with adequate tissue and divided them into two groups according to the expression level of PD-L1. The correlation of PD-L1 expression and the clinicopathological characteristics was evaluated by Pearson Chi-square test. Kaplan-Meier curves was applied to present the probability of survival between PD-L1 expression level and overall survival (OS). Moreover, the literature on the immunotherapy of advanced PPLELC published in PubMed between 2016 and 2020 were reviewed and the efficacy of immunotherapy were analyzed.

**Results:** A total of 18 patients pathologically diagnosed as PPLELC were included. After a follow-up period of 8.8–138 months, 14 patients survived, three patients died and one patient lost, the median OS was 45.3 months Seven samples (tissue-available) tested by NGS and the median TMB was 2.5 mutations/Mb. 19 somatic mutated genes were recognized and TP53 (43%) and CYLD (43%) were the two most commonly mutated genes. Only seven patients who underwent NGS were tested for PD-L1. Three patients with high PD-L1 expression (PD-L1≥ 50%) and four patients with low PD-L1 expression (PD-L1 <50%) were included. No significant correlation was observed between PD-L1 expression and clinical characteristics (age, gender, smoking status, tumor stage, lymph node metastasis) (*p* > 0.05) and OS (*p* = 1). What’s more, 10 PPLELC patients involved in previous studies and one patient received nivolumab in the current study were collected retrospectively. 4/11 (36.4%) patients achieved PR, 6/11 (54.5%) patients achieved SD, and 1/11 (9.1%) patients achieved PD and the disease control rate (DCR) was 90.9%.

**Conclusions:** The prognosis of PPLELC is better than that of other NSCLC, and immunotherapy may be a promising treatment to prolong the survival of advanced PPLELC patients. Whether the immunotherapy efficacy of PPLELC can be predicted by PD-L1 and TMB needs further clinical investigation. CYLD genetic alterations may participate in Epstein–Barr virus-mediated tumorigenesis in PPLELC, providing a novel therapeutic target.

## Highlights

This may be one of the first retrospective study based on the patient data of over a decade to understand the molecular characteristics and immunotherapy reaction of PPLELC. The strength of the work is complimented not only in the results obtained but also in the crisp methodology of the planning and designing of the experiments using simple analytical techniques. Statistical validation/significance of the data may not be adequate as the sample size is very limited.

## Introduction

Lymphoepithelioma-like carcinoma (LELC) is a rare malignant tumor, which shares similar histology with undifferentiated nasopharyngeal carcinoma (NPC). It occurs in the submandibular gland, parotid gland, thymus, lung, stomach, uterus, bladder, and skin (Bégin et al., 1987). Primary pulmonary lymphoepithelioma-like carcinoma (PPLELC) is a rare lung tumor with specific clinicopathological characteristics (Liang et al., 2012), affecting younger patients (51–55-year-old), mostly non-smokers, and mainly Asian females and women from Southern China (Huang et al., 2007; Sun et al., 2014; Jiang et al., 2016). Moreover, a strong correlation has been established between Epstein–Barr virus (EBV) infection and the histological characteristics of PPLELC, which are similar to those of NPC (Huang et al., 2012; Liang et al., 2012).

The majority of the patients with PPLELC do not have obvious clinical manifestations at the time of diagnosis (Grimes et al., 2015), and the treatment modalities of PPLELC follow NSCLC regimens (Ho et al., 2000; Ho et al., 2009). In addition, patients with advanced PPLELC are less likely to benefit from targeted therapy due to the low mutation rates of epidermal growth factor receptor (EGFR), anaplastic lymphoma kinase (ALK), and Kirsten rat sarcoma viral oncogene (KRAS), v-RAF murine sarcoma viral oncogene homolog B1 (BRAF), repressor of silencing 1 (ROS1) and protein 53 (P53) genes (Chang et al., 2011; Liang et al., 2012; Chang et al., 2015). However, the high expression of programmed death receptor ligand-1 (PD-L1) in PPLELC indicates that PD-L1 inhibitors such as nivolumab and pembrolizumab may be suitable treatment options (Chang et al., 2015; Fang et al., 2015; Xie et al., 2018). The efficacy of nivolumab in the treatment of advanced NSCLC is significantly related to tumor mutation burden (TMB), and it is more effective in patients with high TMB compared to chemotherapy (Carbone et al., 2017; Peters et al., 2017). However, although the TMB of PPLELC is low, a large amount of gene copy number variations (CNVs), especially 11q13.3 amplification and 9p21.3 deletion, have been observed (Hong et al., 2019).

**FIGURE 1:**
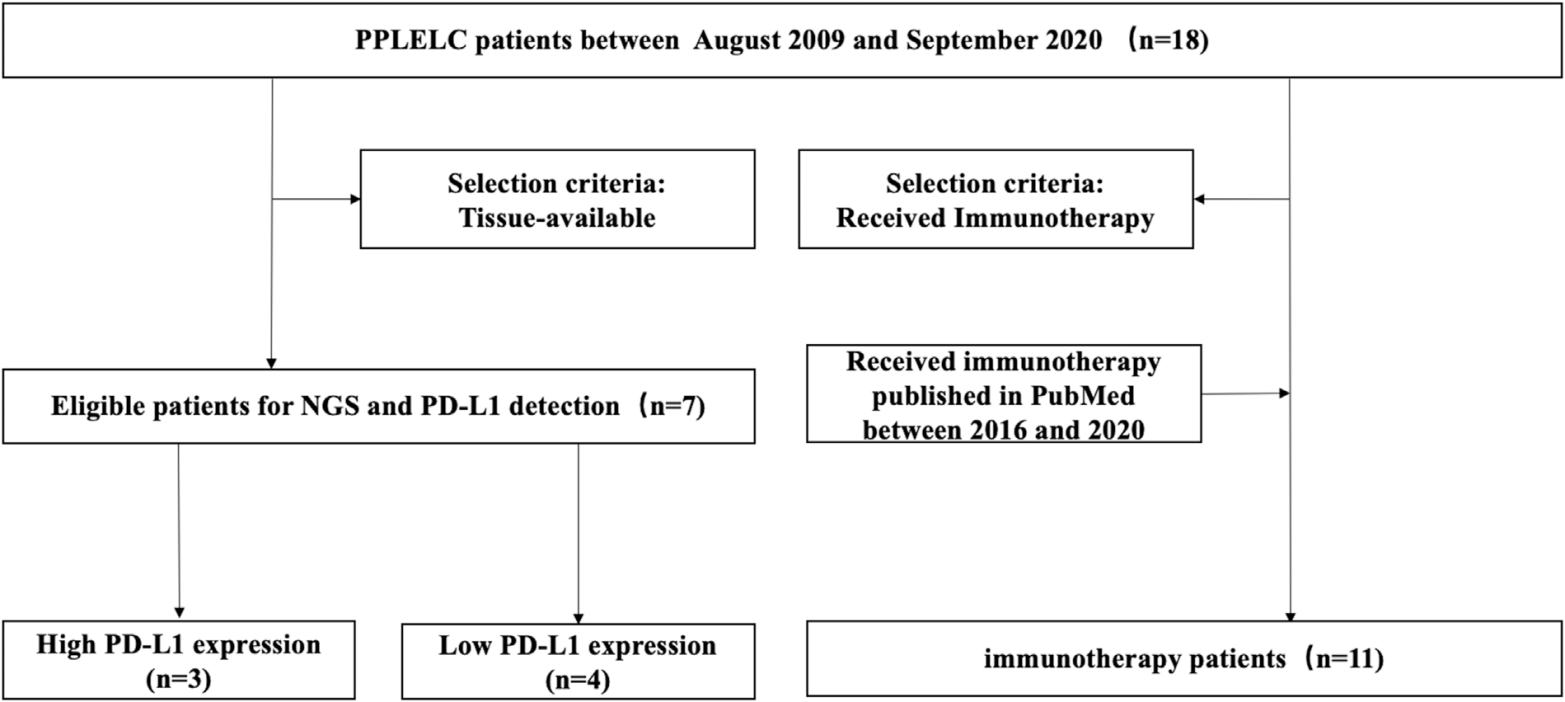
Sample collection and study design of study.

In the present study, we retrospectively collected 18 lung tissue samples of PPLELC diagnosed in our hospital. Next-generation sequencing (NGS) was performed in seven patients who have enough tissue and divided into two groups according to the expression level of PD-L1 to analyze the genetic signature and TMB. Furthermore, the literature on the immunotherapy of advanced PPLELC were reviewed to analyze the efficacy of immunotherapy. The flowchart of the sample collection and study design was shown in Figure 1.

## Materials and Methods

### Sample Collection

Formalin-fixed paraffin-embedded (FFPE) blocks from patients with pathologically confirmed PPLELC in Zhejiiang Cancer Hospital (Hangzhou, China) were enrolled. Those with the second primary malignant tumors (except cervical carcinoma *in situ* and skin basal cell carcinoma) and other primary LELCs outside the lung were excluded. Nasopharyngoscopy or Magnetic Resonance Imaging (MRI) was done to rule out lung metastasis from NPC in all the patients. Medical records were retrieved to collect clinicopathologic data, treatment history and survival outcomes. The follow-up deadline was December 30, 2020.

### Immunohistochemistry Analysis

The specimens were fixed in 10% neutral formalin solution for 24 h and embedded in paraffin. Then the tissue block was cut into 4-μm thick serial sections and baked at 60°C for antigen retrieval. The slices were incubated with the primary antibody at 4°C overnight. Subsequently, the slices were labeled with horseradish peroxidase and stained with chromoplasma matrix to identify the target protein. Phosphate-buffered saline (PBS) was used as a negative control instead of a primary antibody. IHC was performed using the PD-L1 clone 22C3 pharmDx kit. PD-L1 tumor proportion score (TPS) was calculated as the percentage of viable tumor cells with complete or partial membrane staining. TPS was interpreted by a pathologist from commercial vendor. The expression level of PD-L1 was represented by TPS (0–1% as negative, 1–49% as low expression, and ≥50% as high expression) (Wang et al., 2018). The IHC data were scored by two pathologists independently. A total of 100 cells were counted to calculate the proportion of stained cells, and TPS ≥ 1% was defined as positive. PD-L1-positivity was defined when the tumor cells in tissue specimen showed at least 1% PD-L1 expression, while the absence of detection or a TPS of < 1% was considered negative.

### *In-Situ* Hybridization Analysis

Patients with adequate tissue were tested for EBER using ISH according to manufacturer’s instructions. The probe for EBER-1 was supplied by OriGene Technologies, Inc., Tumor nuclei stained with brown granules were interpreted as positive.

### DNA Extraction

From each tumor-rich FFPE and matched normal lung tissue block, 4 μm of sections were cut, deparaffinized, and dissected to isolate 1 cm^2^ of tumor tissue. DNA was isolated using the Cobas R DNA Sample Preparation Kit, according to the manufacturer protocol (Roche Molecular Systems, Pleasanton, CA, United States). The DsDNA concentration was determined using the Qubit R _ 2.0 Fluorometer and the Qubit R 2.0 dsDNA HS Assay Kit (ThermoFisherScientific, Waltham, MA, United States). The quality of the sample DNA was evaluated using a specimen control size ladder test (Invivoscribe Technologies, San Diego, CA, United States).

### NGS

Clinical annotations were extracted from their medical records. Tumor and matched normal DNA were subjected to NGS, and the genomic landscape was explored for potential mutations and therapeutic targets. The genomic information was obtained from NGS-based YuanSu™ 450 gene panel (OrigiMed, Shanghai, China), which encompassed all coding exons of 450 cancer-related genes and 64 selected introns of 39 frequently rearranged solid tumor-related genes. The genes were captured and sequenced with a mean depth of 800X using Illumina NextSeq 500 (Illumina Inc.). Genomic alterations (GAs) were identified by the alignment of sequences from tissues with matched normal lung tissue, as described previously. The TMB was estimated by counting the somatic mutations containing single nucleotide variations (SNVs) and Indels per Mb of the sequence examined in each patient. The driver mutations and recorded germline alterations were not counted.

### Statistical Analysis

Statistical analyses were performed with the R (version 4.1.1). The correlation of PD-L1 expression and prognosis and the clinicopathological characteristics (age, gender, smoking status, lymph node metastasis, and TNM stage) of patients was evaluated by Pearson Chi-square test. Survival curves were plotted using the Kaplan-Meier method and the differences in survival rates were assessed using the log-rank test. Univariate and multivariate analysis of prognostic factors was performed using the Cox proportional hazards model. A *p* value lower than 0.05 (*p* < 0.05) was considered to indicate a significant difference. Somatic mutations in p53 were retrieved from cbioPortal (http://www.cbioportal.org/). Graphs were prepared with “ggplot2” (Wickham, 2009).

### Follow-Up

The follow-up deadline was December 30, 2020. 14 patients were still alive, two patients were deceased and one patient was lost to follow-up. The survival time was counted from the date of pathological diagnosis.

### Search Strategy and Curative Effect Judgment

A comprehensive search was performed through PubMed using the literature retrieval strategy “[pulmonary lymphoepithelioma-like carcinoma (Title/Abstract)] AND [immunotherapy (Title/Abstract)] OR [pulmonary lymphoepithelioma-like carcinoma (Title/Abstract)] AND PD-L1 (Title/Abstract)]” in December 2020 (no year limit and all languages included). Relevant articles were obtained, and references from each of these articles were further searched for relevant articles. A total of 25 articles were reviewed (three were case reports or case series). The clinical efficacy, including complete response (CR), partial response (PR), stable disease (SD), and progressive disease (PD), was evaluated according to the Response Evaluation Criteria in Solid Tumors (RECIST) version 1.1 standard to analyze the efficacy of immunotherapy in patients with advanced PPLELC (Eisenhauer et al., 2009). The objective response rate (ORR), overall survival (OS), and disease-free survival (DFS) were used as the observation indexes. The immune-related adverse reactions (ir-AEs) during the treatment were assessed based on the criteria of common AEs (Common Terminology Criteria for Adverse Events (CTCAE) version 4.0).

## Results

### Clinicopathological Data

A total of 18 cases of PPLELC diagnosed in our hospital from August 2009 to September 2020 were enrolled in this study, and the basic clinical features were summarized in Table 1. Among them, eight patients were men and 10 were women. The patients were middle-aged (average 57 years, range 43–79 years), and 6/18 (33.3%) patients were smokers. 16 patients instead of 18 patients performed an EBER test due to the lack of sufficient tissue of two patients and the expression of Epstein–Barr virus-encoded RNA (EBER) was positive (16/16). 13 patients underwent surgery, of which six received adjuvant platinum-based chemotherapy, four received adjuvant radiotherapy while three did not received any treatment after surgery. Five patients did not receive surgery. Palliative therapy, including concurrent chemoradiotherapy, was undertaken in four patients while one patient did not receive any treatment after diagnosis. As of the follow-up deadline, 14 patients were still alive, two died, and one was lost to follow-up and the median OS was 45.3 months (Supplementary Table S1).

**TABLE 1:** The clinical characteristics of PPLELC (*n* = 18).

Age at diagnosis	Years
Median	57
Range	43–79
Gender	No. of patients
Male	8
Female	10
Smoking situation	No. of patients
Smoker	6
Non-smoker	12
Specimen source	No. of patients
Surgical	13
Biopsies	5
EBER	No. of patients
Positive	16
Negative	0
Clinical stage	No. of patients
I	7
II	2
III	6
IV	3

**FIGURE 2:**
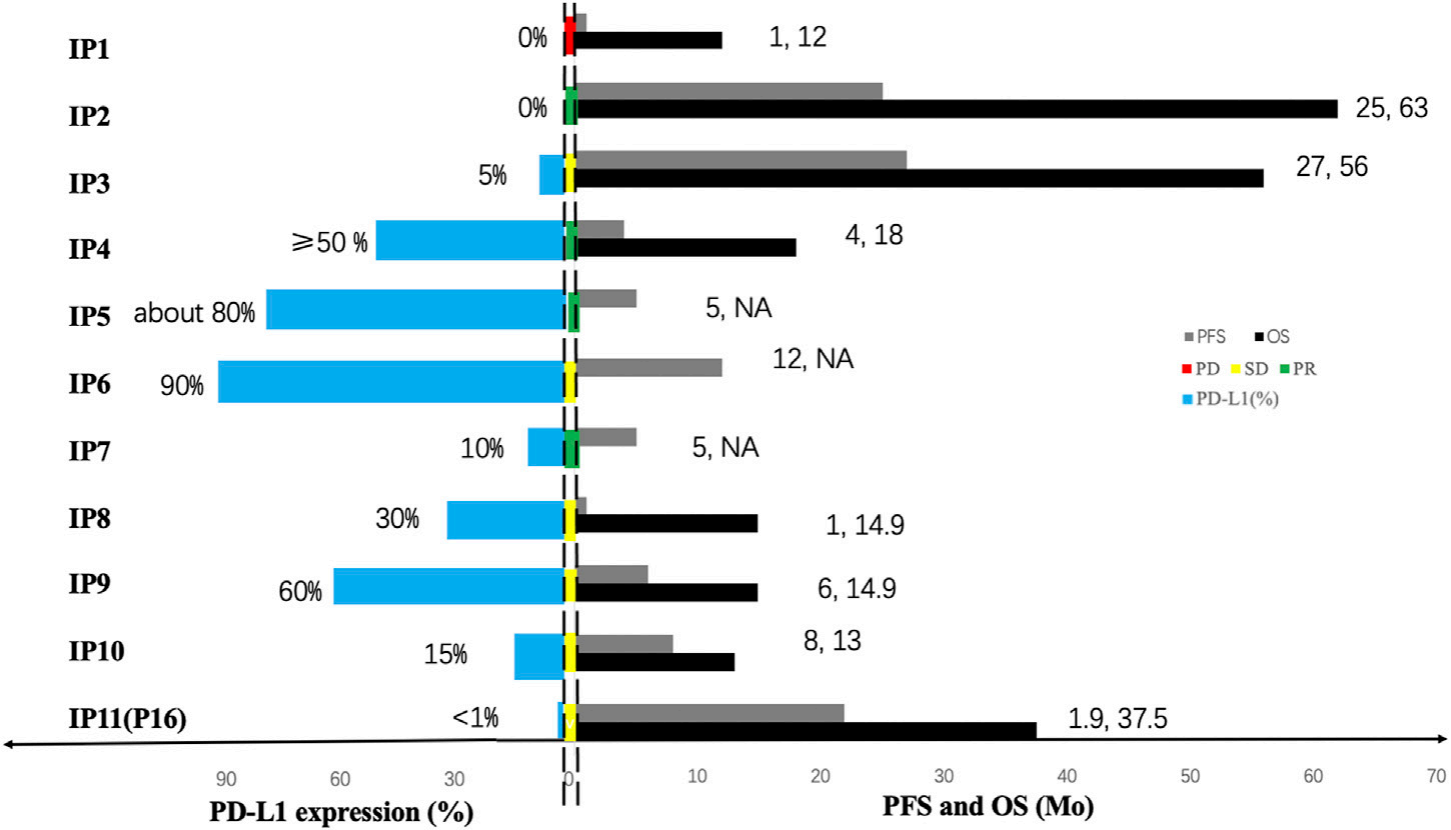
The expression of PD-L1 and immunotherapy reaction of 11 patients. For each immunotherapy patient (marked as “IP”), the bars in the left side of two dotted line represent the expression of PD-L1 and the bars in the middle of two dotted line represent the treatment response to immunotherapy, the green one symbolizes partial response (PR), the yellow symbolizes stable disease (SD) and the yellow symbolizes progressive disease (PD). The bars in the right side of two dotted line represent the prognosis in each patient, black bars and grey bars indicate that the PFS and the OS, respectively. The OS time of two patient was not available and marked as “NA”.

Moreover, the information of 11 immunotherapy patients (10 PPLELC patients involved in previous studies (Kim et al., 2016; Kumar et al., 2017; Narayanan et al., 2019; Qiu et al., 2019; Zhou et al., 2019; Tang et al., 2020; Xie et al., 2020) and one patient received nivolumab in the current study marked as “IP”) were collected retrospectively (Table 2). 4/11 (36.4%) patients achieved PR, 6/11 (54.5%) patients achieved SD, and 1/11 (9.1%) patients achieved PD. The expression of PD-L1 and immunotherapy reaction of 11 patients was shown in Figure 2.

**TABLE 2:** The studies concerning immunotherapy of advanced PPLELC.

Study	No. of cases	Sex; age; smoking	Stage	The expression of PD-L1 (%)	The line of immunotherapy	Immunotherapy	iAEs	Response	Outcome	PFS (mo)	OS (mo)
Kim et al. (2016)	1	F; 37; No	IV (at recurrence)	0	3	Nivolumab	Yes	PD	Died	1	12
Kumar et al. (2017)	2	M; 56; Yes	IV	0	2	Nivolumab	No	PR	Alive	25	62
		F; 37; No	IIIA	5	3	Nivolumab	No	SD	Alive	27	56
Narayanan et al. (2019)	1	F; 76; No	IV	≥50	4	Atezolizumab	No	PR	Died	4	18
Qiu et al. (2019)	1	M; 56; No	IVA	about 80	2	Nivolumab	No	PR	Alive	5	NA
Zhou et al. (2019)	1	F; NM; NA	IV	90	4	Pembrolizumab	NA	SD	NA	12	NA
Tang et al. (2020)	1	F; 50; No	IVB	10	2	Nivolumab	No	PR	NA	5	NA
Xie et al. (2020)	3	F; 56; No	IV (at recurrence)	30	2	Nivolumab	NA	SD	Alive	1	14.9
		F; 49; No	IIIB	60	2	Nivolumab	NA	SD	Alive	6	14.9
		M; 48; No	IVA	15	3	Camrelizumab	NA	SD	Alive	8	13
The current study (P16)	1	M; 57; No	IVA	<1	2	Nivolumab	No	SD	Alive	21.9	37.5

M, male; F, female; iAE, immune-related adverse event; D, died; A, alive; NA, not available; PD, progressive disease; SD, stable disease; CR, complete response; OS, overall survival, mo, month.

### IHC Analysis

All 18 patients were diagnosed as PPLELC based on pathological detection with various lung-cancer-related IHC markers (Table 3). The expressions of IHC markers were highly correlated with the occurrence and progression of PPLELC. The majority of the patients showed positive for P40 (14/15) and CK5/6 (13/14), but negative for thyroid transcription factor-1 (TTF-1) (12/15) and CK7 (8/9). Moreover, all patients were positive for Ki-67 (6/6) and EMA (3/3), while negative for Napsin A (8/8) and CK5/6 (3/3).

**TABLE 3:** The expression of immunohistochemical markers of PPLELC patients.

IHC marker	Positive	Negative
Ki-67	6	0
EMA	3	0
P40	14	1
CK5/6	13	1
P63	11	1
CK	7	1
Napsin A	0	8
CD56	0	3
CK7	1	8
TTF-1	3	12

### Gene Mutation and TMB Analyses

**FIGURE 3:**
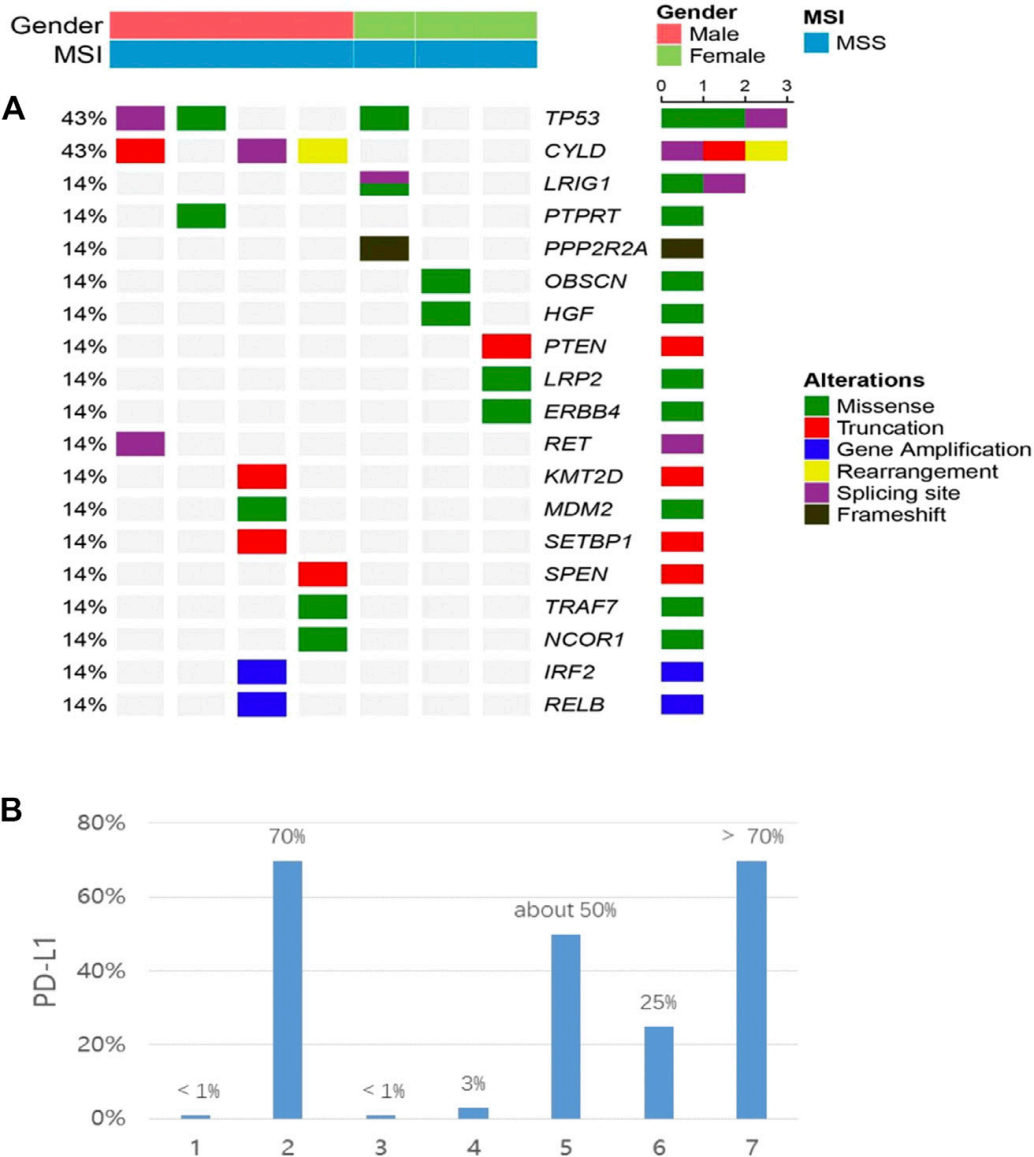
**(A)** co-mutation plot of various types of mutations in all patients. Genes were grouped according to their functions. Each column represents one patient. The mutation rates of each gene were marked on the left in percentage and grouped according to their protein functions. Patient characteristics such as gender, disease stage and tumor type were shown at the top with different colors. **(B)** the expression of PD-L1 in each patient. All patients were placed in the same order in the two panels.

**FIGURE 4:**
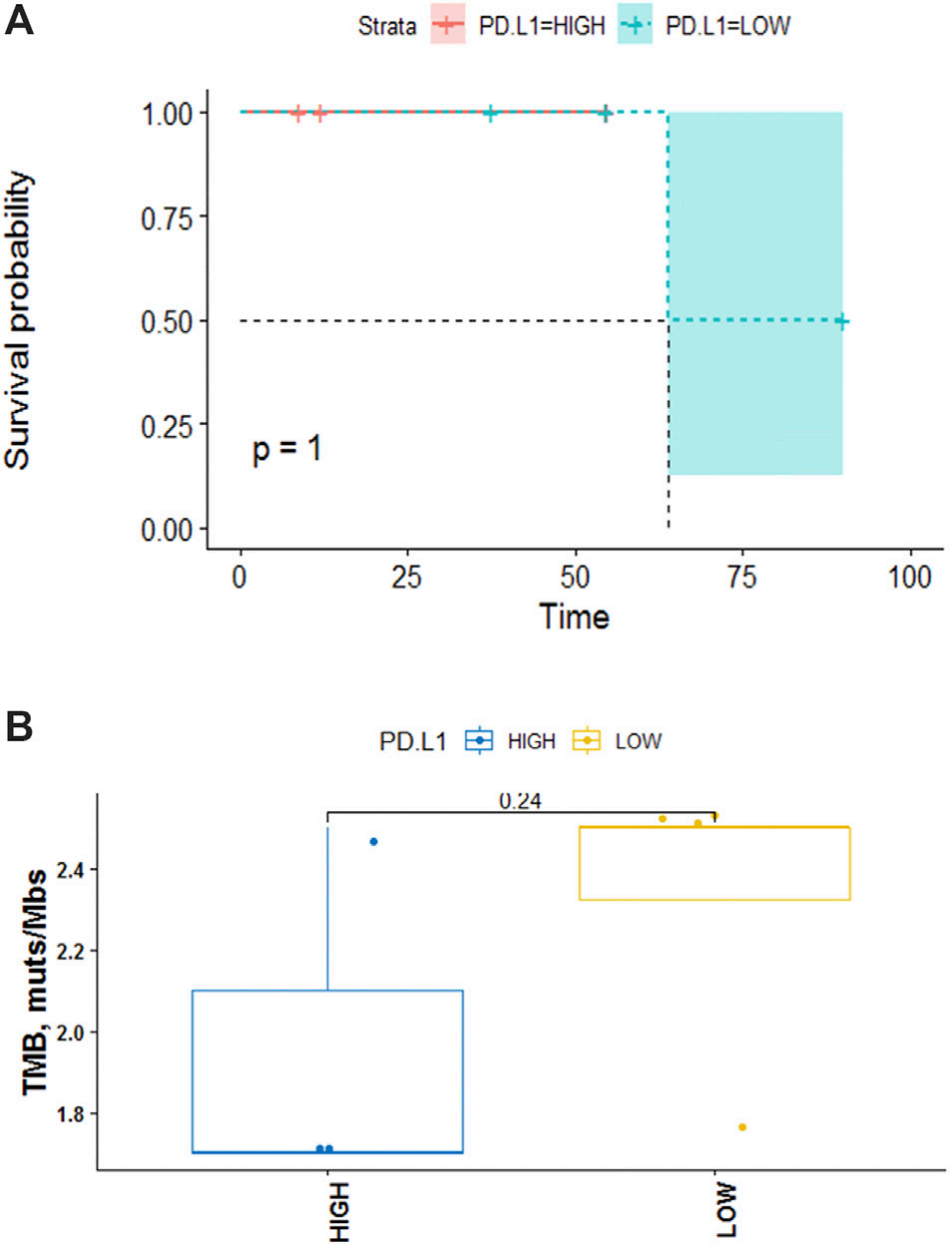
**(A)** Kaplan-Meier analysis of the effect of PD-L1 on OS. The data indicated that no significant correlation between PD-L1 and disease prognosis (*p* = 1). **(B)** TMB in high PD-L1 expression group versus low. The data indicate no significant correlation between the expression level of PD-L1 and TMB in PPLCLC patients (*p* = 0.24).

Seven samples (tissue-available) tested by NGS and the mutation information (include the mutation type, position, functional changes, and so on) was shown in Supplementary Table S2. 19 somatic mutated genes were recognized, TP53 (43%) and CYLD (43%) were the two most commonly mutated genes. A lollipop chart for TP53 is shown in Supplementary Figure S1. Other mutations occurred in LRIG1 (14%), PTPRT (14%), PPP2R2A (14%), and other 17 genes (Figure 3A). The mutation information (include the mutation type, position, functional changes, and so on) was shown in Supplementary Table S3. The median TMB was 2.5 mutations/Mb. The differences of TMB between high and low PD-L1 expression groups were assessed via the Fisher’s exact test and the *p*-value was 1 (Figure 4B).

### The Association Between PD-L1 and Prognosis

Seven patients who underwent NGS were tested for PD-L1. 3/7 (42.8%) patients had ≥50% PD-L1 expression, 2/7 (28.6%) patients showed PD-L1 expression ≥1% and 2/7 (28.6%) patients had **<1%** PD-L1 expression (22C3) (Figure 3B). We defined 50% as the cut-off value and seven patients were divided into two groups. Higher than and/or equal to 50% was considered high (PD-L1≥ 50% as high expression) and lower than 50% low (PD-L1 <50%). No significant correlation was observed between PD-L1 expression and clinical characteristics (age, gender, smoking status, tumor stage, lymph node metastasis) (*p* > 0.05) (Table 4). PD-L1 expression was not associated with OS (*p* = 1) (Figure 4A).

**TABLE 4:** The association between PD-L1 and clinical characteristics.

	High PD-L1 expression	Low PD-L1 expression	*p*-value
	N = 3	N = 4	
Age			
<60	1 (33.3%)	2 (50%)	1
≥60	2 (66.7%)	2 (50%)	
Gender			
Male	1 (33.3%)	3 (75%)	0.741
Female	2 (66.7%)	1 (25%)	
Smoking			
Yes	0	3 (75%)	0.225
No	3 (100.0%)	1 (25%)	
Stage			
I-II	1 (33.3%)	4 (100%)	0.277
III-IV	2 (66.7%)	0	
Lymph node metastasis			
N0	1 (33.3%)	3 (75%)	0.741
N+	2 (66.7)	1 (25%)	

## Discussion

PPLELC has obvious racial and geographical distribution characteristics. Among the 18 patients in our study, the male to female ratio was 4:5, the median age was 57 years, and the ratio of non-smokers to smokers was 2:1, which was consistent with previous studies (Ho et al., 2000; Grimes et al., 2015). However, the epidemiological characteristics of patients in Western countries may differ from those of all Chinese cases. In He et al. (2015) inclusion of 62 cases of the United States’ Open Database was performed; the median age of PPLELC patients was 65 (15∼86) years, with slightly more male than female patients, accounting for 58.1% (36/62). The occurrence of PPLELC was related to EBV infection (Bégin et al., 1987), and EBER test results played a role in the diagnosis of PPLELC. 16 patients were all positive for EBER testing, similar to previous results (Liang et al., 2012; Ma et al., 2013; Mo et al., 2014). EBV infection may be related to race and region. Almost all Asian patients have positive EBV detection, while most non-Asian patients showed negative results (Grimes et al., 2015).

IHC markers are significant in pathological diagnosis, especially for PPLELC, since they are not solely based on the morphologic features. The histopathological features of PPLELC are similar to those of undifferentiated nasopharyngeal carcinoma, and need to be distinguished from metastatic NPC, poorly differentiated primary lung squamous cell carcinoma and lung lymphoma (Da-yun et al., 2017; Anand et al., 2018; Qin et al., 2019). Wang et al. (2013) analyzed the pathological characteristics of 14 patients with PPLELC and pointed out that the high expressions of AE1/AE3, CK5/6, CK19, and LMP-1 could aid in the diagnosis. In the current study, a complete IHC detection was not performed on the patients due to retrospective analysis. However, in the tested patients, the positive rate of squamous cell carcinoma labeled with antibody p63 and CK5/6 was 91.7 and 92.6%, respectively. while the negative rate of TTF-1 and CK7 antibody-labeled adenocarcinoma was 80 and 88.9%, respectively, which were consistent with the above results. PPLELC had a low expression of glandular epithelial markers (TTF-1, CK20, and Napsin-A) and neuroendocrine differentiation markers (CgA, Syn, and CD56), but high expression of squamous epithelial markers (CK5/6, p63, and p40), indicating that PPLELC was derived from epithelial tissue with similar characteristics to squamous epithelial differentiation (Liang et al., 2012).

PPLELC had a better prognosis, and the two and 5-years survival rates were significantly higher than those of the non-LELC patients (both *p* < 0.05) (Han et al., 2001; Qin et al., 2019). In this study, the median DFS (mDFS) of 13 (72.2%) patients who underwent surgery supplemented by chemotherapy and radiotherapy was 45.5 (range 12.2–136) months. Therefore, early surgical resection of PPLELC is feasible, and no lymph node metastasis and complete resection of the tumor could improve the survival rate of the patients (Liang et al., 2012; Lin et al., 2016). Treatment at advanced tumor stages relies on multimodal therapy, including chemotherapy, and radiotherapy. In the present study, platinum-based regimens were our first choice owing to the similarity of PPLELC to NPC (Huang et al., 2007). However, patients with advanced PPLELC are less likely to benefit from targeted therapy. Several studies have explored the role of typical lung carcinogenic pathways in the development of PPLELC and found that advanced PPLELC is less likely to benefit from targeted therapy (Chang et al., 2011; Liu et al., 2014; Chang et al., 2015; Fang et al., 2015; Yeh et al., 2019). In the current study, instead of interrogating only the classic lung cancer oncogenic drivers, we utilized NGS consisting of 450 cancer-related genes and 64 selected introns of 39 solid tumor-related genes that were frequently rearranged to obtain a comprehensive mutation profile of PPLELE. No frequently altered driver genes (e.g., EGFR, KRAS, and BRAF) in classic NSCLC was detected in our cohort which consistent with previous reports.

Instead, the results revealed that TP53 (43%) and CYLD (43%) were the two most commonly mutated genes, and mutations in other 17 genes including LRIG1 (14%), PTPRT (14%), and PPP2R2A (14%) gene were also noted (Figure 2A). A previous study also reported that TP53 mutations E298X, R273C, and G279R were detected in three PPLELC patients, resulting in a TP53 mutation rate of 6.5% (Chang et al., 2011). Notably, the frequency of the TP53 mutation rate was much lower than the result in this study. These findings could be attributed to the following reasons. Firstly, different detection methods produced different proportions of positive cells and different staining intensities. Secondly, increasing the sample size improved the accuracy of the TP53 mutation rate. TP53 mutation could lead to increase in tumor potential gene mutation and PD-L1 expression and may be served as a pair of potential predictive factors in guiding anti-PD-1/PD-L1 immunotherapy (Gibbons et al., 2014; Cortez et al., 2015). TP53 or KRAS mutation patients significantly prolonged PFS compared with wild-type patients who underwent pembrolizumab treatment (mPFS, TP53-mut vs. KRAS-mut vs. wild-type: 14.5 vs. 14.7 vs. 3.5 months *p* = 0.012) (Dong et al., 2017). Yin L et al. found that the presence of CYLD enhanced the chemosensitivity of bladder cancer to gemcitabine (Yin et al., 2016). Additionally, the curative effect of gemcitabine combined with cisplatin was significantly better compared to pemetrexed combined with cisplatin for PPLELC (*p* < 0.001) (Hong et al., 2019) indicating that the presence of CYLD in PPLELC may also enhance the chemosensitivity of gemcitabine. The efficacy of gemcitabine in the treatment of EBV-related tumors could be improved by ganciclovir (Feng et al., 2004), and the efficacy of ganciclovir combined with gemcitabine in the treatment of PPLELC needs further exploration.

PD-L1 expression is higher in PPLELC compared to conventional NSCLCs (Fang et al., 2014; Chang et al., 2015), assuming that it is a potential biomarker and rational therapeutic target. All PPLELC patients expressed PD-L1, including 42.6% (3/7) high expression and 57.1% (4/7) moderate expression (Figure 3B). The high expression of PD-L1 in PPLELC sheds light on the possibility of using immunotherapy in this subtype of lung cancer. The up-regulation of PD-L1 expression may be related to EBV infection and EBV-related tumors may be more beneficial in the treatment of immune checkpoint inhibitors (Jiang et al., 2015). However, there are few study on the comparison of immunological efficacy differences between EBV-related and non-EBV-related malignancies. 11 cases of advanced-stage PPLELC progressed continually despite multiple lines of chemotherapy but responded favorably to a PD-L1 inhibitor and the disease control rate (DCR) was 90.9% (Figure 2). Noteblely, two patients with -negative PD-L1 (<1%) received immunotherapy, one patient achieved PD, while another patient achieved PR. The PD-L1-negative patient (PD-L1 < 1%) also received immunotherapy and responded to nivolumab for 48 cycles that lasted 21.9 months, indicating a long-term tumor response of PD-1/PD-L1 inhibitors in patients irrespective of PD-L1 status (Brahmer et al., 2015). Therefore, only a minority of patients acquired a good response to immunotherapy, although it could be highly effective. The effectiveness of immunotherapy may not only depend on the expression of PD-L1 in tumor tissue but also on whether there are sufficient immune effector cells in the tumor microenvironment (TME) (Zhang and Chen, 2016). A large number of lymphocytes with CD8^+^ and TIA-1+ and cytotoxic T cells were detected, and plasma cell infiltration occurred in the stroma around the PPLELC tumor cells (Qin et al., 2019; Sathirareuangchai and Hirata, 2019). This might be one of the reasons why PPLELC patients benefit from immunotherapy despite the low expression of PD-L1.

The high nonsynonymous TMB was associated with improved objective response, durable clinical benefit, and progression-free survival (PFS) after immunotherapy (Rizvi et al., 2015; Devarakonda et al., 2018). Additionally, previous data showed that the median TMB of patients with PPLELC was 1.6 mutations/Mb, which was significantly lower than that of patients with lung adenocarcinoma in the TCGA data set of cancer genome map (*p* < 0.01) (Xie et al., 2020; Cancer Genome Atlas Research Network, 2014). Herein, the median TMB of our cohort was 2.5 (range 1.7–2.5 mutations/Mb, Figure 4B). Therefore, TMB could not fully reflect the immunogenicity of the tumor. Patients with low TMB could also respond to immunotherapy, while patients with high TMB may not have a good effect, which depends on human leukocyte antigen (HLA) classification (Chowell et al., 2018). The more diversity of HLA, especially the more super-subtypes of HLA-B44, the more kinds of new antigens can be presented, and the better the efficacy of immune drugs will be improved. However, Mcgranahan et al. (2017) confirmed that patients with clonal HLA heterozygote deletion had higher mutation levels and obvious subclonal expression of tumor cells in NSCLC patients compared to HLA patients without heterozygote mutation. Although their TMB levels were high, the immunotherapy was ineffective. Therefore, TMB and PD-L1 could complement each other to predict the efficacy of immunotherapy. Yet, no correlation between low/high PD-L1 expression and TMB value was observed (Fisher’s exact test, *p* = 0.24) which need to be verified by additional prospective studies.

There are several limitations. The sample size was small and this was retrospective study. Due to its rare incidence, only 18 patients were diagnosed with PPLELC at our center over the last decade and only seven patients performed NGS. Statistical validation/significance of the data may not be adequate as the sample size is very limited. Second, immunotherapy is a relatively new treatment method for PPLELC. 11 advanced PPLELC patients who received immunotherapy were analyzed retrospectively with different regimens, aimed to demonstrate that the use of immune checkpoint may be promising beneficial treatments. Whether heterogeneity exists in the treatments used also requires further exploration. Multicenter studies with sufficiently long observation periods will be carried out in the future to provide more evidence.

In summary, PPLELC is a rare subtype of NSCLC which is closely related to EBV. Surgery is the first choice of treatment for early diagnosed patients, while and radiotherapy/chemotherapy can prolong the survival of advanced PPLELC patients. Although EGFR-sensitive mutations and other classical lung cancer gene mutations are rare, however, CYLD may be a new therapeutic target for PPLELC. Despite the low TMB, the PD-L1 positivity of a majority of the tumors raises the potential of utilizing checkpoint immunotherapy as a treatment regimen that could benefit PPLELC patients. However, the mechanism of immune checkpoint blockade in PPLELC remains yet unclear, and the activity of immune checkpoint inhibitors observed in other virus-associated cancers warrants further evaluation of this new class of cancer therapeutics in patients with PPLELC.

## Data Availability

The data that support the findings of this study have been deposited into CNGB Sequence Archive (CNSA) of China National GeneBank DataBase (CNGBdb) with accession number CNP0002253.
