# Supplementary material for: Molecular and Clinical Characteristics of Primary Pulmonary Lymphoepithelioma-Like Carcinoma
Source: Front Mol Biosci. 2021 Oct 25;8:736940. doi: 10.3389/fmolb.2021.736940 (PMC8573970; doi:10.3389/fmolb.2021.736940)
Supplement: Supplementary file 1 [file Table1.DOCX]

**Supplementary Table 1** The treatment modalities and prognosis of PPLELC patients

| **Patient ID** | **Stage** | **NACT** | **Surgery** | **CT** | **RT** | **IT** | **Survival status** | **OS (mo)** |
| --- | --- | --- | --- | --- | --- | --- | --- | --- |
| **P1** | IA1 | No | Yes | No | No | No | Alive | 27.4 |
| **P2** | IA1 | No | Yes | Yes (PC, 4 cycles) | No | No | Alive | 45.3 |
| **P3** | IA2 | No | Yes | No | No | No | Alive | 28.5 |
| **P4** | IA2 | No | Yes | No | No | No | Lost | 54.6 |
| **P5** | IA3 | No | Yes | No | No | No | Dead | 64 |
| **P6** | IA3 | No | Yes | No | No | No | Alive | 90.1 |
| **P7** | IB | No | Yes | No | No | No | Alive | 12.2 |
| **P8** | ypIIB | Yes (TP, 2 cycles) | Yes | Yes (TP, 2 cycles) | No | No | Alive | 12.5 |
| **P9** | IIB | No | Yes | No | No | No | Alive | 136 |
| **P10** | ypIIIA | Yes (GP, 4 cycles) | Yes | Yes (GP, 4 cycles) | Yes | No | Alive | 45.3 |
| **P11** | ypIIIA | Yes (TP, 3 cycles) | Yes | Yes (TP, 2 cycles) | Yes | No | Alive | 45.5 |
| **P12** | ypIIIA | Yes (DP, 4 cycles) | Yes | - | No | No | Alive | 56.1 |
| **P13** | IIIA | No | Yes | Yes (NP, 2 cycles; AP, 2 cycles) | Yes | No | Alive | 55 |
| **P14** | IIIB | No | No | Yes (GP, 1 cycle) | Yes | No | Alive | 17.7 |
| **P15** | IIIB | No | No | No | No | No | Dead | 23.2 |
| **P16** | IVA | No | No | Yes (TP, 6 cycles) | No | Yes | Alive | 37.5 |
| **P17** | IVA | No | No | Yes (TP, 4 cycles) | No | No | Alive | 8.8 |
| **P18** | IVB | No | No | Yes (VP-16, QD*10) | Yes | No | Dead | 28.7 |

NACT neoadjuvant chemotherapy, CT chemotherapy, RT Radiotherapy, IT immunotherapy, OS overall survival, mo months, PC pemetrexed plus carboplatin, TP Paclitaxel plus cisplatin/carboplatin, GP gemcitabine plus cisplatin/carboplatin, DP docetaxel plus cisplatin, NP vinorelbine plus cisplatin
