# Supplementary material for: Molecular and Clinical Characteristics of Primary Pulmonary Lymphoepithelioma-Like Carcinoma
Source: Front Mol Biosci. 2021 Oct 25;8:736940. doi: 10.3389/fmolb.2021.736940 (PMC8573970; doi:10.3389/fmolb.2021.736940)
Supplement: Supplementary file 2 [file Table2.DOCX]

**Supplementary Table 2**: The mutation information of 7 PPLELC patients

| **Patient ID** | **Gene** | **Alternation type** | **Chromosome** | **Position start** | **Coding DNA change** |
| --- | --- | --- | --- | --- | --- |
| **1 (P6)** | CYLD | Truncation | chr16 | 50785686 | c.676G>T |
|  | RET | Substitution | chr10 | 43598087 | c.625+10G>A |
|  | TP53 | Splice site | chr17 | 7579591 | c.97-1G>T |
| **2 (P7)** | CYLD | Substitution | chr20 | 40944545 | c.1957C>T |
|  | RET | Substitution | chr17 | 7577509 | c.772G>A |
| **3 (P16)** | CYLD | Splice site | chr16 | 50816378 | c.1826+1G>C |
|  | IRF2 | Gene Amplification | - | - | - |
|  | KMT2D | Truncation | chr12 | 49425644 | c.12844C>T |
|  | MDM2 | Substitution | chr12 | 69218420 | c.512T>C |
|  | RELB | Gene Amplification | - | - | - |
|  | SETBP1 | Truncation | chr18 | 42531785 | c.2480G>A |
| **4 (P5)** | CYLD | Rearrangement | - | - | - |
|  | NCOR1 | Substitution | chr17 | 16001745 | c.2756C>T |
|  | SPEN | Truncation | chr1 | 16258127 | c.5392C>T |
|  | TRAF7 | Substitution | chr16 | 2225398 | c.1483G>A |
| **5 (P17)** | *LRIG1* | Substitution | chr3 | 66457771 | c.1957C>T |
|  | *LRIG1* | Substitution | chr3 | 66457907 | c.772G>A |
|  | *PPP2R2A* | Splice site | chr8 | 26151240 | c.1826+1G>C |
|  | *TP53* | Gene Amplification | chr17 | 7577506 | - |
| **6 (P4)** | HGF | Truncation | chr7 | 81336668 | c.12844C>T |
|  | OBSCN | Substitution | chr1 | 228462037 | c.512T>C |
| **7 (P13)** | ERBB4 | Gene Amplification | chr2 | 212570100 | - |
|  | LRP2 | Truncation | chr2 | 170094614 | c.2480G>A |
|  | PTEN | Rearrangement | chr10 | 89720743 | - |
