# Supplementary figures and images for: Molecular and Clinical Characteristics of Primary Pulmonary Lymphoepithelioma-Like Carcinoma
Source: Front Mol Biosci. 2021 Oct 25;8:736940. doi: 10.3389/fmolb.2021.736940 (PMC8573970; doi:10.3389/fmolb.2021.736940)

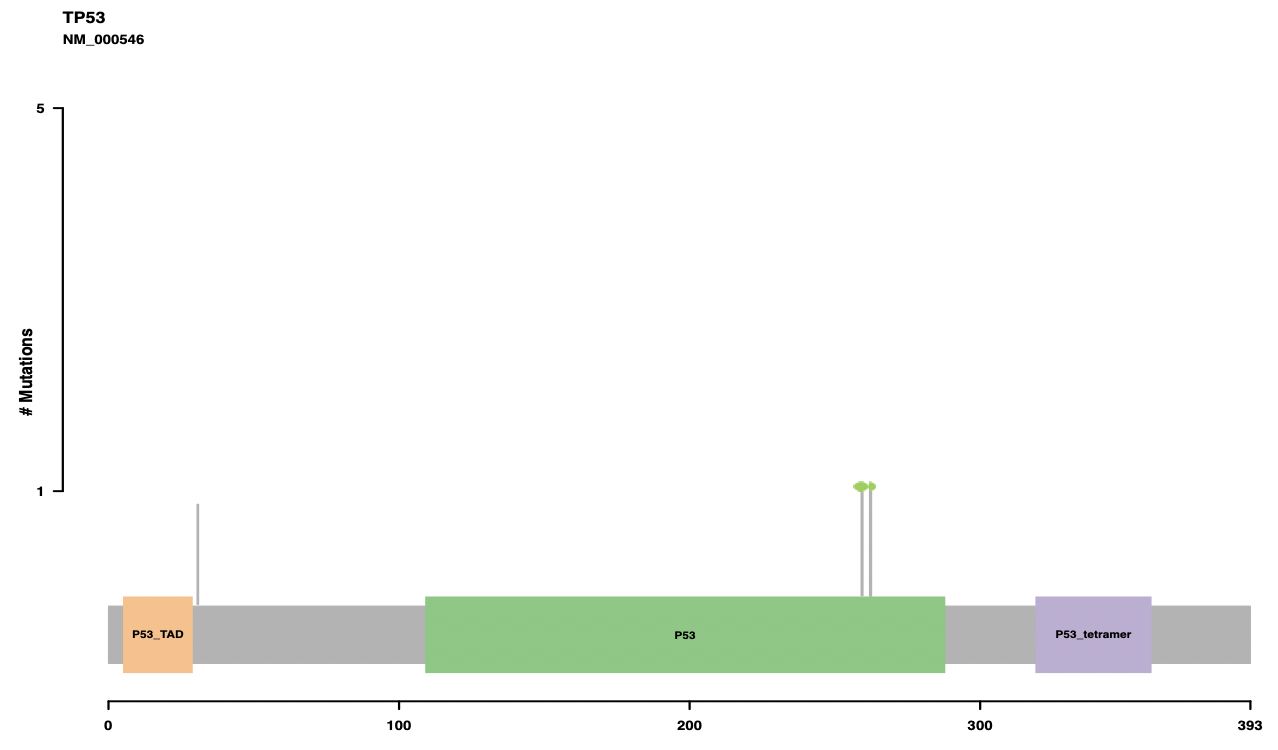

Supplement: Supplementary file 3 [file Image1.PNG]
